# Co-evolution of epidemiology and artificial intelligence: challenges and opportunities

**DOI:** 10.1093/ije/dyad089

**Published:** 2023-06-22

**Authors:** Joohon Sung, John L Hopper

**Affiliations:** Genome and Health Data Laboratory and Department of Epidemiology, Graduate School of Public Health, Seoul National University, Seoul, Korea; Institute of Health and Environment, Seoul National University, Seoul, Korea; Centre for Epidemiology and Biostatistics, School of Population and Global Health, University of Melbourne, Melbourne, VIC, Australia

## Legacy of epidemiology

Epidemiology, as suggested by its etymology, has been dedicated to discovering knowledge that can improve the health of populations. Epidemiology has evolved as a discipline that combines qualitative and quantitative designs and techniques to identify true and likely causal signals against a background of confounding and random errors. From the examples of tobacco smoking regulation to COVID-19 guidelines, epidemiological evidence has contributed to the identification of proper intervention targets, so as to have an impact on the population's health.

## The big data era and a burgeoning of data science and artificial intelligence (AI)

Artificial intelligence (AI), also often referred to as machine learning (ML) and deep learning (DL) (see Box 1 for clarification), is an automated process whereby information is extracted from a given dataset using computing techniques to create an algorithm for making predictions and/or classifications.^1^ The key difference between AI and classic epidemiology is that the latter builds models based on explicit assumptions about what matters and how, so that the results can be directly interpretable, whereas AI builds algorithms in essence for predictive models discovered from the data, without necessarily understanding why. The trend toward larger and more complex ‘big’ datasets (see Box 1) is inevitable and irreversible. Big data unfortunately suffer from major problems. For example, the shift in electronic health records (EHR) data over time which accompanies the evolution of clinical practice, the sparse density arising from ever-increasing data points, and the inclusion of unstructured data, are but a few of the challenges. Novel AI approaches are increasingly being required to properly process data silos into an analysis-ready format so as to handle the complexity and abundance of data.

Box 1 Confusing terms and terms with different meaning in epidemiology, statistics and artificial intelligence

**Artificial intelligence (AI), machine learning (ML) and deep learning (DL):**

AI emulates human intelligence systems by using computers. ML is a subtype of AI that can improve prediction algorithms using data with ‘ground truth’ (answers). Note the differences with conventional programming, which uses ‘pre-completed’ algorithms (= rule-based methods) to get answers. DL is a specific type of ML that uses deep layers of neural networks inspired by human neurons. Although the majority of modern successes of AI are attributable to DL, AI is widely used as a lay term to mean both DL and ML with a long history. In this article, we use the term AI instead of DL.

**Big data:**

Big data refers to datasets that are too large (in amount) or complicated (in structure) to be dealt with by traditional data processing methods using well-defined database programs (e.g. sorting, merging and taking representative values). By ‘big’, the size of observations is generally more important, contrasting with so-called ‘fat data’ with fewer observations relative to the number of variables in datasets. From the epidemiology angle, big data is also characterized by lacking prior analytical plans but later utilized for research purposes; electronic health records (HER) from hospital administration and lifelogs from smartphone use are typical examples.

**Bias**:

In epidemiology, bias means systematic errors in statistical inference which arise from weaknesses in study designs and conduct. In statistical inference, bias is defined as a difference between the expected value of an estimate and its true value. Statistical analyses cannot correct biases due to faulty design. In AI, bias means the intercepts in unit models (i.e. *bi* of yi = ai * Xi + bi).

**Parameter, parametric and hyperparameter:**

In statistics, a parameter refers to the characteristic of the population, such as the mean, variance or some other aspect of the probability distribution of one or more random variables. A parametric model is one in which the relationship between random variables is assumed to follow a particular equation. In AI, a parameter refers to the components that AI learns from model training. Hyperparameter in AI refers to values needed to be set using a priori insight or experience. Regularization terms or batch size are hyperparameters in DL.

**Cause, causality, and association**

In epidemiology, cause and causality are reserved for associations verified at a biological level or by experimental studies. Otherwise, all potential causal relations are defined as associations, recognizing that these could be due to causation in one of both directions or by uncontrolled confounding. In statistical models, an association refers to the result with statistical significance rejecting the null hypothesis, although statistical parlance often refers to these associations as ‘effects’, which unfortunately implies association despite the caveats above. AI generally fails to distinguish between cause and association because it is mainly interested in prediction, irrespective of the reason why. In AI, features with high ‘feature importance’ are generally considered meaningful predictive factors. Causal or counterfactual models in AI usually denote generative adversarial networks (GAN) where the other label (= outcome in epidemiology) is replaced in training and comparing the results between the original and reversed models.

**Model development and validation:**

In epidemiology, after the development of an explanatory model, validation is conducted using an independent dataset; that is, there is a distinction between internal and external validation depending on the source of the validation dataset. In AI, model development is denoted as ‘training’, and for validation, mixing training and validation subsets from the same dataset commonly occur (e.g. iterated k-fold crossvalidation). Such validation is internal. For this reason, AI generally uses a third ‘test’ process with a strictly independent subset from the same dataset.

**‘Fairness’, ‘fairness of algorithm’, and ‘fairness of data’**

In ML, predictions made by an ML algorithm are considered unfair if the performance of the predictions depends on variables considered to be ‘sensitive.’ (such as race and sex) Unfair algorithms would result in both quality and equity problems. Data scientists attempt to assure algorithmic fairness based on ‘correction algorithms’. The ‘metrics’ of fairness are, in essence, the differences in the predictive performance (e.g. positive or negative predictive accuracy) between the groups divided by sensitive traits. The unfairness of data is, if not entirely, responsible for ML's algorithmic unfairness, given ML's overfitting abilities. This ‘fairness’ issue applies to all sub-populations, however they be defined, and would result in unsatisfactory performances of the resultant algorithms. The ‘Dictionary of Epidemiology’^22^ lacks the term ‘fairness’. Semantically similar ‘equity’ is one of the major interests in epidemiology, if not a key concept of its discipline. However, fairness in ML is more about the equality of metrics than bioethics. From an epidemiology angle, thus fairness is more related to data representativeness without selection and information bias. Epidemiology has unique strengths and an established discipline to understand and handle those biases.

## Successful examples and unintended consequences of AI in health

When applied to health, AI has had numerous successes, including diagnostic support for image analysis^2^^,^^3^ and automated interpretation of echocardiography and electroencephalography.^4^^,^^5^ On the other hand, unintended consequences and unsatisfactory performances have also been reported. One primary problem lies in that AI-based methods will not work for ‘minority’ sub-groups, however defined, given they have been under-represented in the training data used for building AI algorithms.

We believe it is essential to take a balanced view of the success and limitations of AI in health, especially in these formative years, by understanding AI's fundamental (intrinsic) versus controllable (extrinsic) problems. We should avoid making undue criticism of AI, because no methods are free from errors.

## Intrinsic versus extrinsic problems of AI in health

AI algorithms derive accuracy metrics based on features extracted from a given dataset, regardless of their causal contributions; see Box 1 and Table 1 for the definition of terms related to AI modelling. The optimization process of AI is highly efficient, but overfitting is inevitable. Diagnostic performance is achieved at the cost of transparency and causality. AI also loses generalizability when the training dataset does not represent the characteristics of the population to which the results are applied (targets), which is the origin of ‘unfairness’ for minority groups. These issues are intrinsic to AI.

**Table 1:** Comparative terminology between epidemiology and artificial intelligence (AI) for similar concepts described by different terms

Epidemiology and/or biostatistics	Artificial intelligence (AI)	Meaning
Measures assessed by a ‘reference standard’ (previously ‘gold standard’) method	Ground truth or label	Actual outcome measurements
Risk factors (epi) or independent variables (stat)	Features	Potential predictor measurements
Model fitting	Model training	Learning the association between predictors and outcome
Internal validation	Validation	Testing the prediction using initial dataset
External validation	Test	Testing the prediction using independent dataset
Prediction	Classification	Performance of the model in terms of risk discrimination
Winner’s curse: (overfitting when extrapolating the results)	Overfitting during training (an inevitable and necessary, intermediate phenomenon in AI)	Model performance outside the learning data is worse than within the dataset

Extrinsic problems of AI stem from the interactions between AI tools and humans. Health practice relies on multidisciplinary experts whose routine is structured into tight schedules where precise and efficient communication matters. In theory, extrinsic problems should be controllable, but technical hurdles to humanizing the AI interface often precipitate real-world problems, impairing the anticipated effectiveness of AI.

## Key epidemiological principles to redress AI’s intrinsic weaknesses in health

Epidemiological discipline has evolved through confronting health problems and customizing practice using well-designed studies, and should now use this experience in the application of AI. Epidemiology has led health research by being continually enriched by other disciplines, and we present ideas on how the same could apply when using AI.

### Providing causal insights

AI is particularly powerful for diagnosis and prediction. However, features with good predictive power do not necessarily have causal relevance, as has been established by theoretical work^6^ and practical examples^7^; see Box 1 for more details about the concept of causality in AI and epidemiology. Application of AI to the recent quasi-experimental methods for making causal inference could make these approaches more powerful.^8^ These methods include: (i) use of directed acyclic graphs (DAGs) when the analysis includes variables that might exert effects on both exposures and outcomes of interest (so-called ‘collider’), or confounding variables that require conditioning^9^; (ii) Mendelian randomization that in theory combines biological knowledge with statistical inference^10^; and (iii) Inference on Causation from Examination of FAmiliaL CONfounding (ICE FALCON) which learns by studying the changes in the regression coefficients before and after conditioning when applied to twin and sibling data.^11^

### Assuring the comparative effectiveness

One fundamental question is whether application of AI to health will improve outcomes and reduce costs. AI solutions need to be tested in the settings where they are to be applied, not just in the controlled settings of an intermediate process used to internally evaluate diagnostic performance.^12^^,^^13^ AI’s intrinsic overfitting is problematic when applied to new data with unseen heterogeneity. Recently, expert groups have been gathering consensus on new reporting guidelines specific to different phases of clinical research involving AI, e.g. TRIPOD-AI for prediction model and development of diagnostics,^14^^,^^15^ DECIDE-AI for early-stage clinical studies,^16^ CONSORT-AI and SPIRIT-AI for clinical trials.^17^

### Assessing the fairness of AI

Algorithmic fairness, defined as the dependency of results or performance on ‘sensitive variables’ such as race or gender, is well-known in the data science society^18^; see Box 1 for clarification of the fairness and related concepts. Multiple software packages for ‘fairness corrections’, such as ‘Fairlearn’ (Microsoft)^19^ and ‘ML-Fairness Gym’ (Google),^20^ have been developed and generally assess the predictive performance metrics between ‘sensitive variables’ and try to reduce the imbalance within a given dataset. This attempt at mitigation invariably results in a mechanical balance of the metrics at the expense of destroying the concept of population inference from random samples.

Epidemiology has long developed theories and remedies for biases that arise from differential selection/participation and heterogeneity.^21^ The fairness of the algorithms would be better evaluated if the differences in frequency between population and sample were estimated and used (e.g. using matching) rather than trying to produce one omnibus solution. Fairness of algorithms and data is another area where epidemiological thinking could contribute, but this has so far largely been neglected by epidemiologists.

## *IJE* efforts and editorial plans

This Editorial is a call for papers in the *IJE* on development and issues arising from the use of artificial intelligence (AI) in epidemiology, and vice versa. The big data era presents both challenges and opportunities for epidemiology. The *IJE* has a keen interest in this brisk change that epidemiology is confronting. A critical evaluation of AI in epidemiological practice, or the need for a new evolution, has already been published in *IJE* and other epidemiology journals.^23^^–^^25^ The authors are keen to consolidate the constructive discussions so far, and suggest topics toward which next discussions might converge. These include, but are not limited to:

- study designs that maximize the informativity and credibility derived from big data;
- causal analysis methods combined with AI analysis;
- proper evaluation of AI solutions for diagnostics and ultimate outcomes;
- roles of epidemiology in assuring fairness and equity of data algorithms derived from big data;
- regulatory or reporting guidelines for studies using AI.

These two planets are getting ever closer. Epidemiologists and AI researchers are beginning to work together, rather than against each other, and respect each other’s disciplines. We envisage the *IJE* initiative to publish papers on this issue will facilitate the co-evolution of epidemiology and AI for the population's health to become the true beneficiary.
